# Validation of the Spanish Childhood Trauma Questionnaire-Short Form in adolescents with suicide attempts

**DOI:** 10.3389/fpsyg.2024.1378486

**Published:** 2024-07-09

**Authors:** Ainoa García-Fernández, Clara Martínez-Cao, Alberto Sánchez-Fernández-Quejo, Teresa Bobes-Bascarán, Jorge Andreo-Jover, Wala Ayad-Ahmed, Ana Isabel Cebriá, Marina Díaz-Marsá, Nathalia Garrido-Torres, Sandra Gómez, Ana González-Pinto, Iria Grande, Noelia Iglesias, Katya B. March, Diego J. Palao, Iván Pérez-Díez, Natalia Roberto, Miguel Ruiz-Veguilla, Alejandro de la Torre-Luque, Iñaki Zorrilla, Víctor Pérez, SURVIVE Group, Pilar A. Sáiz, María Paz García-Portilla

**Affiliations:** ^1^Department of Psychiatry, Universidad de Oviedo, Oviedo, Spain; ^2^Instituto de Investigación Sanitaria del Principado de Asturias (ISPA), Oviedo, Spain; ^3^Instituto Universitario de Neurociencias del Principado de Asturias (INEUROPA), Oviedo, Spain; ^4^Department of Legal Medicine, Psychiatry, and Pathology, Universidad Complutense de Madrid (UCM), Madrid, Spain; ^5^Servicio de Salud del Principado de Asturias (SESPA), Oviedo, Spain; ^6^Department of Psychology, Universidad de Oviedo, Oviedo, Spain; ^7^Hospital La Paz Institute for Health Research (IdiPAZ), Madrid, Spain; ^8^Department of Psychiatry, Universidad Autónoma de Madrid (UAM), Madrid, Spain; ^9^Hospital Clínico San Carlos, Madrid, Spain; ^10^CIBER de Salud Mental, Instituto de Salud Carlos III, Madrid, Spain; ^11^Mental Health Service, Hospital Universitari Parc Taulí, Unitat Mixta de Neurociència Traslacional (I3PT-INc-UAB), Sabadell, Barcelona, Spain; ^12^Department of Clinical and Health Psychology, Faculty of Psychology, Universitat Autònoma de Barcelona, Cerdanyola del Vallès, Spain; ^13^Institute of Biomedicine of Seville (IBiS), Sevilla, Spain; ^14^Hospital Virgen del Rocío, Sevilla, Spain; ^15^Department of Psychiatry, University of Seville, Sevilla, Spain; ^16^Departament de Medicina, Facultat de Medicina i Ciències de la Salut, Institut de Neurociències, Universitat de Barcelona (UB), Barcelona, Spain; ^17^Bipolar and Depressive Disorders Unit, Hospìtal Clinic de Barcelona, Barcelona, Spain; ^18^Institut d’Investigacions Biomèdiques August Pi i Sunyer (IDIBAPS), Barcelona, Spain; ^19^Department Psychiatry, UPV/EHU, Vitoria, Spain; ^20^BIOARABA, Vitoria, Spain; ^21^Hospital Universitario de Alava, Vitoria, Spain; ^22^Department of Psychiatry, Clinical Psychology and Mental Health, La Paz University Hospital, Madrid, Spain; ^23^Department of Psychiatry and Forensic Medicine, Faculty of Medicine, Universitat Autònoma de Barcelona, Cerdanyola del Vallès, Spain; ^24^Department of Psychiatry, Universidad Autónoma de Madrid (UAM), Madrid, Spain; ^25^Institute of Neuropsychiatry and Addictions, Hospital del Mar, Barcelona, Spain; ^26^Department of Psychiatry and Forensic Medicine, Autonomous University of Barcelona, Barcelona, Spain; ^27^Hospital del Mar Medical Research Institute, Barcelona, Spain

**Keywords:** CTQ-SF, reliability, validity, suicide attempt, adolescent

## Abstract

**Background:**

Child maltreatment is associated with a higher probability of mental disorders and suicidal behavior in adolescence. Therefore, accurate psychometric instruments are essential to assess this.

**Objective:**

To validate the Spanish version of the Childhood Trauma Questionnaire-Short Form (CTQ-SF) in adolescents with suicide attempts.

**Methods:**

Multisite cohort study of 208 adolescents with suicide attempts using data from the following scales: Mini International Neuropsychiatric Interview (MINI), Columbia Suicide Severity Rating Scale (C-SSRS), Patient Health Questionnaire (PHQ-9), and CTQ-SF. Statistical analysis: CTQ-SF scores analyzed by descriptive statistics. Internal consistency: McDonald’s omega and Cronbach’s alpha. Concurrent validity with PHQ-9 and C-SSRS scores: Spearman correlation coefficient. Structural validity: Confirmatory factor analysis.

**Results:**

Floor and ceiling effects: Physical abuse and neglect as well as sexual abuse demonstrated high floor effects (50.0, 35.1, and 61.1% of adolescents, respectively). No ceiling effects were found. The CTQ-SF had excellent internal consistency (McDonald’s omega = 0.94), as did the majority of its subscales (Cronbach’s alpha 0.925–0.831) except for physical neglect (0.624). Its concurrent validity was modest, and the emotional neglect subscale had the lowest Spearman correlation coefficients (0.067–0.244). Confirmatory factor analysis: Compared with alternative factor structures, the original CTQ-SF model (correlated 5-factor) exhibited a better fit [S-B *χ*^2^ = 676.653, *p* < 0; RMSEA (90% CI = 0.076–0.097) = 0.087; SRMR = 0.078; CFI = 0.980; TLI = 0.978].

**Conclusion:**

The Spanish CTQ-SF is a reliable, valid instrument for assessing traumatic experiences in adolescents at high risk of suicide. It appears appropriate for use in routine clinical practice to monitor maltreatment in this group.

## 1 Introduction

Child maltreatment encompasses all forms of physical and emotional ill-treatment, sexual abuse, neglect, and exploitation that result in actual or potential harm to the child’s health, development, or dignity (World Health Organization, 2022). Exposure to such traumatic experiences in childhood may affect proper neurobiological, cognitive, and affective development in these individuals (Pérez-Balaguer et al., 2022). This unfortunate reality makes them part of a vulnerable group, increasing their susceptibility to developing psychosocial problems if they are not adequately protected (Georgieva et al., 2021). Additionally, childhood maltreatment is linked to a variety of mental disorders as well as an increase in suicide attempts (Chen et al., 2021). Except for emotional neglect, all childhood maltreatment subtypes are associated with non-suicidal self-injury (NSSI) (Liu et al., 2018). Therefore, accurate detection and evaluation of these experiences is essential to understanding and preventing the negative consequences that can arise as a result of exposure to traumatic situations.

Among the available instruments for the retrospective assessment of childhood maltreatment, the Childhood Trauma Questionnaire-Short Form (CTQ-SF) is one of the most widely used and validated instruments to measure and assess traumatic experiences during childhood and adolescence (Saini et al., 2019; Georgieva et al., 2021). Originally developed from an initial 70-item version by Bernstein et al. (1994) and Bernstein and Fink (1998), this self-report questionnaire measures several types of traumatic experiences quantitatively. Furthermore, Bernstein et al. (1997) replicated their previous results in the adolescent psychiatric population and provided initial support for the validity of the CTQ in this population as well. Five years later, Bernstein et al. (2003) developed a short form of the CTQ (the CTQ-SF), providing a briefer approach to measuring trauma. Additionally, it is supported by strong evidence and has been translated into multiple languages and applied across diverse populations worldwide (Saini et al., 2019; Malan-Muller et al., 2022). Some investigators, such as Hernandez et al. (2013), have referred to it as the “gold standard” for the assessment of maltreatment in childhood. The evidence for the CTQ-SF factorial structure is controversial. While several research groups confirmed the standard 5-factor structure of the CTQ-SF (Dovran et al., 2013; Karos et al., 2014; Charak et al., 2017; Sacchi et al., 2018; He et al., 2019; Petrikova et al., 2021), others supported it only partially or suggested alternative models to the original structure (Gerdner and Allgulander, 2009; Kim et al., 2011; Grassi-Oliveira et al., 2014; Kongerslev et al., 2019; Aloba et al., 2020; Behn et al., 2020). Remarkably, the physical neglect subscale is problematic in these studies. Given these inconclusive research results, further efforts to examine its factor structure are essential.

CTQ-SF internal consistency was good, ranging from 0.79 to 0.94, and comparable across previously published studies (Gerdner and Allgulander, 2009; Kim et al., 2011; He et al., 2019; Kongerslev et al., 2019; Petrikova et al., 2021). Again, the physical neglect subscale had the poorest internal consistency of all five scales in most studies. Moreover, some studies also tested and demonstrated measurement invariance based on sex (Charak et al., 2017; He et al., 2019; Aloba et al., 2020), age (Grassi-Oliveira et al., 2014) and population type, e.g., substance abuse, inmate, psychiatric, and adolescent samples (Dovran et al., 2013).

Hernandez et al. (2013) conducted an initial validation of the Spanish version of the CTQ-SF in an adult female clinical sample (mean age 41.6 years) undergoing psychiatric outpatient (48%) or inpatient treatment. They demonstrated adequate psychometric properties and a good fit of the 5-factor structure, replicating the original study’s findings. As in the CTQ-SF English version, the physical neglect scale showed the lowest internal consistency and factor loadings. However, no studies have used this questionnaire to examine childhood maltreatment in the Spanish adolescent population at high risk of suicide and the relation of these adverse experiences with suicidal behaviors. Therefore, this study aims to validate the Spanish version of the self-reported CTQ-SF and assess its psychometric properties, including reliability, validity, and factorial structure, in a clinical sample of adolescents with suicide attempts.

## 2 Materials and methods

### 2.1 Participants

In all, 208 adolescents who had completed the Spanish version of the CTQ-SF (Hernandez et al., 2013) were included in the study. All patients were recruited at the psychiatric emergence department from seven University Hospitals across Spain: Hospital Clinic (*n* = 49) and Corporació Sanitària Parc Taulí (*n* = 38) in Barcelona; Hospital Clínico San Carlos (*n* = 18) and Hospital Universitario La Paz (*n* = 49) in Madrid; Hospital Universitario Central de Asturias (*n* = 34) in Oviedo; Hospital Universitario Araba-Santiago (*n* = 19) in Vitoria, and Hospital Universitario Virgen del Rocío (*n* = 1) in Seville. Inclusion criteria were: (1) females or males, (2) between 12 and 18 years old, (3) informed consent of the patient who made the suicide attempt, (4) informed consent of their parents or legal guardians, and (5) undergoing outpatient care (Psychiatrist and/or Clinical Psychologist) in the Child and Youth Mental Health Centers or Child and Youth Day Hospital. Exclusion criteria were: (1) incapacity to give informed consent, (2) lack of fluency in Spanish, and (3) currently participating in another clinical study likely to interfere with this study.

### 2.2 Instruments

Trained psychologists assessed the participants within 10 days following the adolescents’ admission to the emergency department due to a suicide attempt. The assessment included an *ad hoc* questionnaire for collecting socio-demographic (age, sex, current academic year) and clinical data (psychiatric diagnosis, depressive symptoms and medical treatment). The Spanish versions of the following instruments were also used: the Mini International Neuropsychiatric Interview for Children and Adolescents (MINI-KID; Sheehan et al., 2010) to assess the presence of psychiatric diagnosis in children and adolescents, and the Columbia Suicide Severity Scale (C-SSRS) (Posner et al., 2011; Al-Halabí et al., 2016) to assess the suicide risk severity.

In addition, the adolescents completed the following self-report scales: Patient Health Questionnaire (PHQ-9) (Diez-Quevedo et al., 2001; Kroenke et al., 2001) to assess depressive symptoms and CTQ-SF.

The CTQ-SF is an instrument designed to explore childhood abuse history. It consists of 28 items, of which 25 are grouped into five subscales: emotional abuse, physical abuse, sexual abuse, emotional neglect, and physical neglect. The remaining items constitute a validity scale (items 10, 16, and 22). Each item is rated on a 5-point Likert-type scale from 1 (never) to 5 (almost always). Some of the items are written in reverse (shown in Table 1 as item number followed by “R”), so those must be recoded before calculating the scores (5 = 1 and 1 = 5). There is a total score and independent scores for the five subscales. Therefore, the total score ranges from 25 to 125 and the subscale scores from 5 to 25. According to the original manual, the cut-off values of the CTQ-SF subscales classify severity into four levels, from “none” to “extreme” (Bernstein et al., 2003).

**Table 1:** Reliability characteristics of CTQ-SF subscales.

CTQ-SF subscale	Cronbach’s alpha	Item number	Mean (SD)	Item-total correlation
Emotional abuse	0.831	3	3.043 (1.404)	0.570
		8	3.000 (1.447)	0.677
		14	2.788 (1.439)	0.632
		18	3.269 (1.492)	0.634
		25	3.168 (1.534)	0.599
Physical abuse	0.845	9	1.370 (0.969)	0.423
		11	2.067 (1.402)	0.606
		12	1.798 (1.328)	0.514
		15	1.961 (1.303)	0.602
		17	1.466 (1.124)	0.418
Sexual abuse	0.925	20	1.908 (1.385)	0.568
		21	1.485 (1.133)	0.464
		23	1.692 (1.333)	0.507
		24	1.865 (1.458)	0.543
		27	1.798 (1.406)	0.510
Emotional neglect	0.848	5R	2.687 (1.485)	0.457
		7R	2.634 (1.293)	0.677
		13R	2.423 (1.309)	0.545
		19R	2.668 (1.307)	0.501
		28R	3.081 (1.303)	0.605
Physical neglect	0.624	1	1.379 (0.825)	0.406
		2R	2.366 (1.297)	0.511
		4	1.519 (1.081)	0.376
		6	1.399 (0.839)	0.405
		26R	1.730 (1.139)	0.659
Validity	0.618	10R	3.355 (1.314)	0.131
		16R	3.865 (1.200)	0.580
		22R	3.557 (1.313)	0.637

CTQ-SF, Childhood Trauma Questionnaire – Short Form; R, inverse item recodified; SD, standard deviation.

### 2.3 Procedure

This secondary analysis for validating a self-assessment instrument for childhood trauma is part of a larger nationwide project called SURVIVE (Survive Prevention and Intervention: cohort study and nested randomized controlled trials of secondary prevention programs for suicide attempts). The SURVIVE project, described in detail by Pérez et al. (2023), encompasses a multi-site cohort study conducted by several research groups across Spain. The recruitment period was from January 2021 to March 2023.

Participants were recruited from psychiatric emergency departments due to a suicide attempt in the 10 days prior to evaluation. All eligible participants were invited to complete an assessment administered by trained psychologists. Both participants and their parents were required to be willing and able to comply with the study procedures and to provide informed consent.

It was approved by the Clinical Research Ethics Committee at each of the participating sites [Hospital Clinic, IDIBAPS (PI19/000954), Hospital Universitario Parc Taulí (PI19/01484), Hospital Clínico San Carlos (PI19/01256), La Paz Institute for Health Research (PI19/00941), Hospital Universitario Central de Asturias (HUCA) (IP19/01027), Hospital Universitario Araba-Santiago (PI19/00569), and Hospital Universitario Virgen del Rocío (PI19/00685)]. It follows national and international guidelines: the latest version of the World Medical Association (2013), developed as ethical principles for medical research involving human subjects, and the International Council for Harmonization (ICH) Good Clinical Practice Guideline, GCP Directive 2005/28/EC. This study also complies with current Spanish legislation: Organic Law 3/2018 of December 5 on the Protection of Personal Data and Guarantee of Digital Rights and Regulation (EU) 2016/679 of the European Parliament and of the Council, version of April 27, 2016, known as the General Data Protection Regulation (GDPR). All participants and their parents or legal guardians gave their written informed consent before enrolment.

### 2.4 Data analysis

The statistical analysis was done using SPSS 27.0 and the software R (version 4.2.1). All tests were two-tailed and the significance level was set at 0.05.

To measure the shape of the distributions, skewness (asymmetry) and kurtosis (peakedness or flatness) were computed. Values of ±1 were considered good. The coefficient of variation (standard deviation/mean) and ceiling and floor effects were also determined [floor and ceiling effects were calculated for each subscale, and the widely used 15% threshold was adopted for patients achieving the highest and lowest scores (Terwee et al., 2007)]. All data were checked for normal distribution with the Kolmogorov–Smirnov test. The data did not meet the normality assumption; thus, non-parametric statistical methods were used for the analyses.

Regarding validity, we assessed concurrent and structural validities. The criterion validity could not be evaluated since no established gold standard is available for comparison with the instrument under evaluation.

An exploratory factor analysis and a principal component analysis was performed in addition, which can be found in the Supplementary material.

Structural validity of the CTQ-SF was tested using confirmatory factor analysis (CFA) with the following packages: psych (Revelle, 2023) and lavaan (Rosseel, 2012).

Given the inconclusive results for the factor structure of the CTQ-SF reported by Sacchi et al. (2018), three competitive factor models were tested: a unifactorial model, a correlated 5-factor model, and an uncorrelated 5-factor model. Prior to the CFA, the 3 validity items (10, 16, 22) were excluded. To evaluate the fit of these models, the weighted least squares (WLS) estimation method was used, as the items were ordinal Likert scale and non-normally distributed data. For these CFA models, factor loadings were freely estimated, the factor variances were fixed to 1, and correlations between factors were allowed. The model fit was evaluated with the Satorra-Bentler Scaled Chi-Square test (S-B *χ*^2^) and associated degrees of freedom (*df*). A good fit is indicated by non-significant S-B *χ*^2^ values and *χ*^2^/*df* coefficients lower than 2 (Newcomb, 1994). Since the S-B *χ*^2^ test is susceptible to sample size, robust versions of fit indices were used to evaluate the different models, including the Comparative Fit Index (CFI), Tucker-Lewis Index (TLI), the root mean square error of approximation (RMSEA), and standardized root mean square residual (SRMR). According to Hooper et al. (2008), appropriate criteria for these indices are as follows: RMSEA <0.08, CFI > 0.95, SRMR <0.08, and TLI > 0.95.

Concurrent validity was examined using the Spearman’s Rho correlation coefficient between the CTQ-SF subscale scores and the PHQ-9 total score as well as the following items of the C-SSRS: number of NSSI and number of suicide attempts (total, interrupted, and aborted attempts).

Concerning reliability, the internal consistency of the overall CTQ-SF was measured using MacDonald’s omega value (ω), which was more appropriate for multidimensional scales (Crutzen and Peters, 2017). Additionally, Cronbach’s alpha coefficients were calculated to evaluate the internal consistency of each subscale.

## 3 Results

### 3.1 Sample

A total of 208 adolescents with suicide attempts were included. The mean age was 15.03 (SD = 1.49), and 87.0% were female. Table 2 shows the demographic and clinical characteristics of the adolescents.

**Table 2:** Demographic and clinical characteristics.

Age [Mean (SD)]	15.03 (1.49)
Sex, female [*n* (%)]	181 (87.0)
**Current academic year [*n* (%)]**	
Primary education	2 (1.0)
1st secondary	16 (7.7)
2nd secondary	32 (15.4)
3rd secondary	47 (22.6)
4th secondary	52 (25.0)
1st bachelor	23 (11.1)
2nd bachelor	17 (8.2)
Vocational training	15 (7.2)
Dropped out of school	4 (1.9)
Have you repeated any grades? Yes [*n* (%)]	53 (25.5)
Suicide attempts: Yes [*n* (%)]	208 (100)
**Total suicide attempts [*n* (%)]**	
Any suicide attempt (1–4)	185 (88.9)
Multiple suicide attempts (5 or more)	23 (11.1)
Non-suicidal self-injury: yes [*n* (%)]	160 (76.9)
Interrupted attempts: yes [*n* (%)]	75 (36.1)
Aborted attempts: yes [*n* (%)]	97 (46.6)
Familial history of attempted suicide: yes [*n* (%)]	53 (25.5)
**Diagnosis according to the MINI: yes [*n* (%)]**	
Major depressive episode	122 (58.7)
Dysthymic disorder	17 (8.2)
Suicide risk	176 (84.6)
Manic episode	4 (1.9)
Mood dysregulation disorder	23 (11.1)
Panic disorder	44 (21.2)
Agoraphobia	28 (13.5)
Separation anxiety	11 (5.3)
Social anxiety	36 (17.3)
Specific phobia	16 (7.7)
Obsessive compulsive disorder	9 (4.3)
Post-traumatic stress disorder	22 (10.6)
Alcohol abuse	6 (2.9)
Alcohol dependence	2 (1.0)
Drug abuse (not alcohol)	6 (2.9)
Drug dependence (not alcohol)	4 (1.9)
Tic disorder	2 (1.0)
Attention deficit disorder with hyperactivity	20 (9.6)
Conduct disorder	7 (3.4)
Oppositional defiant disorder	5 (2.4)
Psychotic disorders	5 (2.4)
Anorexia nervosa	24 (11.5)
Bulimia nervosa	29 (13.9)
Generalised anxiety disorder	58 (27.9)
Adjustment disorder	37 (17.8)
PHQ-9 total score [Mean (SD)]	18.39 (5.24)
**Pharmacological treatment: yes [*n* (%)]**	
Antidepressants	126 (60.6)
Antipsychotics	61 (29.3)
Benzodiazepines	65 (31.3)

MINI, Mini International Neuropsychiatric Interview; PHQ-9, Patient Health Questionnaire; SD, standard deviation.

### 3.2 Psychometric properties of the CTQ-SF

#### 3.2.1 Distribution characteristics and descriptive statistics of CTQ-SF scores

The distribution characteristics of the CTQ-SF are shown in Table 3. In all cases, the mean scores exhibit no normality of distribution (Kolmogorov–Smirnov test <0.05). Only two subscales (emotional abuse and emotional neglect) show symmetrical distributions and platykurtic kurtosis, with more scattered values and fewer data in the central region. The other three subscales show an asymmetric distribution, with values >1, and a more concentrated data distribution around the mean. The physical abuse and neglect subscales and the sexual abuse subscale demonstrate a striking floor effect (50.0, 35.1, and 61.1% of adolescents, respectively). On the contrary, none of the subscales show a ceiling effect (values between 0.0 and 9.1% of adolescents).

**Table 3:** Descriptive analysis of CTQ-SF subscale scores.

CTQ-SF subscale	Mean (SD)	Range	Skewness (SE)	Kurtosis (SE)	Normality test, *p*	Floor effect (%)	Ceiling effect (%)
Emotional abuse	15.26 (5.65)	5–25	−0.110 (0.169)	−0.951 (0.336)	0.075, 0.007	8.2	9.1
Physical abuse	8.66 (4.85)	5–25	1.493 (0.169)	1.411 (0.336)	0.227, 0.000	50.0	1.0
Sexual abuse	8.75 (5.91)	5–25	1.530 (0.196)	1.060 (0.336)	0.290, 0.000	61.1	4.3
Emotional neglect	13.49 (5.28)	5–25	0.138 (0.169)	−0.909 (0.336)	0.073, 0.009	11.5	2.4
Physical neglect	8.36 (3.32)	5–19	1.177 (0.169)	0.866 (0.336)	0.188, 0.000	35.1	0.0

CTQ-SF, Childhood Trauma Questionnaire – Short Form; SD, standard deviation; SE, standard error.

#### 3.2.2 CTQ-SF validity

##### 3.2.2.1 Structural validity

Results of the CFA show that the classic correlated 5-factor model provides a good fit with the observed data [S-B *χ*^2^ = 676.653, *p* < 0; RMSEA (90% CI: 0.076–0.097) = 0.087; SRMR = 0.078; CFI = 0.980; TLI = 0.978]. Although the *χ*^2^ statistic is significant and the *χ*^2^/*df* ratio is above 2, these outcomes might be influenced by the limited sample size (Kenny and McCoach, 2003). On the contrary, the other 2-factor solutions resulted in unsatisfactory models, suggesting that modifications of the original 5-factor structure were not necessary (see Table 4).

**Table 4:** Fit indices for CTQ-SF models.

Models	*df*	SB-*χ*^2^ (*p*-value)	*χ*^2^/*df*	RMSEA [90% CI]	SRMR	CFI	TLI
One general factor	275	3031.621 (<0)	11.024	0.220 [0.211, 0.229]	0.173	0.868	0.856
Five uncorrelated factors	275	6985.483 (<0)	25.402	0.343 [0.336, 0.351]	0.323	0.679	0.650
Five correlated factors	265	676.653 (<0)	2.553	0.087 [0.076, 0.097]	0.078	0.980	0.978

CTQ-SF, Childhood Trauma Questionnaire – Short Form; *df*, degree of freedom; SB-*χ*^2^, Satorra-Bentler Scaled Chi-square test; RMSEA, Root Mean Square Error of Approximation; SRMR, Standardised Root Mean Square Residual; CFI, Comparative Fit Index; TLI, Tucker-Lewis Index.

**Figure 1:**
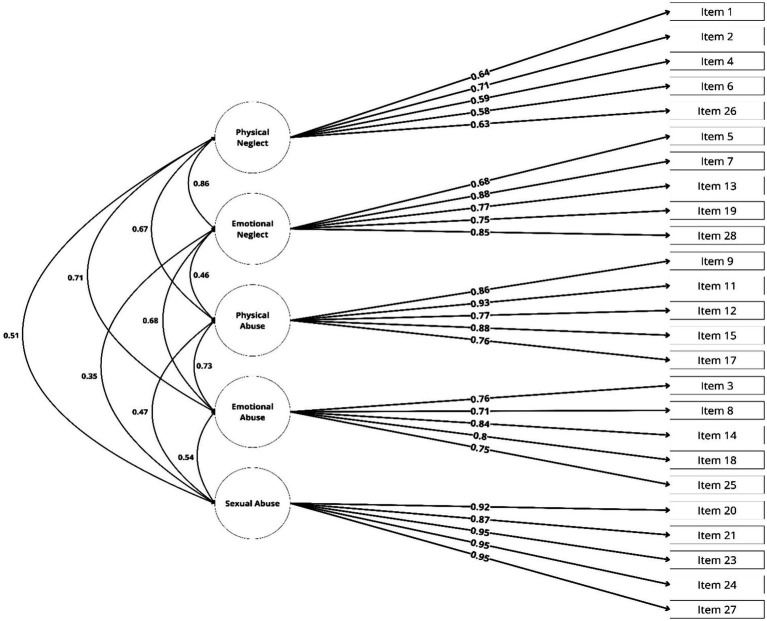
Confirmatory factor analysis of the original structure of CTQ-SF with items distributed into five standard subscales. CTQ-SF, Childhood Trauma Questionnaire – Short Form.

All items had factor loadings higher than 0.6 with the exception of items 4 and 6 (0.589 and 0.578, respectively) (see Table 5). Therefore, the results of this analysis support an underlying structure of five components in this sample (Figure 1). On the other hand, as expected, these five components are intercorrelated, with correlation coefficients ranging from 0.857 between emotional neglect and physical neglect to 0.346 between emotional neglect and sexual abuse (see Supplementary Table S2).

**Table 5:** Standardized factor loadings for CTQ-SF based on confirmatory factor analysis.

Items	Factor loading
**Sexual abuse**	
Item 20	0.92
Item 21	0.869
Item 23	0.945
Item 24	0.947
Item 27	0.953
**Emotional abuse**	
Item 3	0.761
Item 8	0.708
Item 14	0.839
Item 18	0.803
Item 25	0.749
**Physical abuse**	
Item 9	0.865
Item 11	0.930
Item 12	0.767
Item 15	0.882
Item 17	0.764
**Emotional neglect**	
Item 5	0.677
Item 7	0.879
Item 13	0.767
Item 19	0.747
Item 28	0.849
**Physical neglect**	
Item 1	0.641
Item 2	0.711
Item 4	0.589
Item 6	0.578
Item 26	0.632

CTQ-SF, Childhood Trauma Questionnaire – Short Form.

##### 3.2.2.2 Concurrent validity

Table 6 shows the correlation coefficients between the original five CTQ-SF subscale scores with the PHQ-9 total score and the C-SSRS items: number of NSSI and number of suicide attempts (total, interrupted, and aborted attempts). Additionally, we are showing the correlation coefficients using our new CTQ-SF subscales. As seen in Table 6, depression scores and the number of suicide attempts significantly correlate with almost all types of maltreatment. In contrast, the number of self-injuries and the subtypes of suicidal behaviors correlate with the CTQ-SF subscales to a lesser extent.

**Table 6:** Correlations among CTQ-SF subscales and other measures.

			# Suicide attempts		
CTQ-SF subscales	PHQ-9 total score	# NSSI	# Total attempts	# Interrupted attempts	# Aborted attempts
Emotional abuse	0.387^**^	0.188^**^	0.297^**^	0.170*	0.208**
Physical abuse	0.185**	0.054	0.263**	0.144*	0.100
Sexual abuse	0.227**	0.192**	0.243**	0.098	0.179**
Emotional neglect	0.244**	0.181**	0.113	0.109	0.067
Physical neglect	0.225**	0.134	0.204**	0.139*	0.118

CTQ-SF, Childhood Trauma Questionnaire – Short Form; NSSI, non-suicidal self-injury; PHQ-9, Patient Health Questionnaire. ^**^Correlation is significant at the 0.01 level. ^*^Correlation is significant at the 0.05 level.

#### 3.2.3 CTQ-SF reliability

The CTQ-SF scale demonstrates excellent internal consistency in adolescents with suicide attempts, with a total Omega of (ωT) = 0.94. On the other hand, all subscales showed good internal consistency, except for physical neglect and the validity items where it is moderate (Cronbach’s alpha of 0.624 and 0.618, respectively) (see Table 1). All but item 10R has corrected item-total correlation values >0.3, ranging from 0.376 (item 4) to 0.677 (items 7R and 8) (see Table 1).

## 4 Discussion

This study investigated the psychometric properties of the Spanish version of the CTQ-SF in Spanish adolescents who made at least one suicide attempt. The results confirm that this version has acceptable psychometric properties.

Regarding the descriptive analysis of its subscale scores, the distribution on three subscales (physical abuse and neglect subscales and sexual abuse subscale) is positively skewed, meaning that higher values are scattered on the right-hand side of the distribution. It should be noted that none of the subscales presented a ceiling effect; however, physical abuse and neglect and sexual abuse showed an enormous floor effect, so the discriminative capacity of these items is limited in that range. Moreover, we found that emotional abuse and emotional neglect were the most common types of traumatization reported by our adolescents, while physical neglect had the lowest occurrence. In other words, almost 70% (*n* = 140) of our sample reported a history of moderate to extreme emotional abuse. This proportion is nearly 50% (*n* = 71) for emotional neglect. In contrast, physical neglect had a higher proportion of participants at lower severity levels (none to minimal). These same results were previously obtained in a similar cultural environment (Reus, Spain) but in an adult female clinical sample (Hernandez et al., 2013).

We explored both the classic and alternative structures of the CTQ-SF in a sample of Spanish adolescents. In comparison with competitive factor models, the classic CTQ-SF model exhibited the best fit to the data, and the original 5-factor model showed reasonable fit (Bernstein et al., 1997).

Additionally, our confirmatory factor analysis results indicated adequate loadings of all items. However, the items belonging to the physical neglect subscale obtained the lowest values. As observed in previous validation studies, the physical neglect subscale has been the most controversial of the instrument. According to Grassi-Oliveira et al. (2014), neglect is a challenging construct to operationalize, as most definitions rely on personal perceptions of a lack of care. More specifically, child rearing practices exhibit considerable variations across cultures. Consequently, it is crucial to investigate whether the interpretation of the items differs among cultures (Grassi-Oliveira et al., 2014) and varies depending on the language spoken.

The directions of the correlations between the CTQ-SF and other study instruments were modest in magnitude but consistent with the expected patterns, confirming its concurrent validity and usefulness as a clinical tool to assess the impact of different types of child maltreatment. Compatible with previous evidence (Aloba et al., 2020), all types of child maltreatment were correlated with the severity of depressive symptoms. This highlights the direct relationship between adverse childhood experiences and the development of mental health problems throughout life, particularly depressive symptoms (Grossberg and Rice, 2023). Additionally, four of the five subscales of the CTQ-SF positively correlated with total suicide attempts. This finding suggests that different types of child maltreatment have a substantial impact on the risk of suicidal behaviors, emphasizing the need to identify and support individuals with a history of child maltreatment to prevent tragic outcomes. Emotional neglect was the sole subscale not associated with any suicidal events (interrupted, aborted, or suicide attempts), but significantly correlated with NSSI. In contrast, emotional abuse was the sole subscale significantly correlated with all suicidal behaviors and with NSSI. The fact that emotional neglect is not associated with suicide attempts but is associated with NSSI suggests that different types of maltreatment may have different effects on mental health outcomes. On the other hand, emotional abuse shows a strong correlation with both suicidal behaviors and NSSI, indicating that this type of mistreatment may have a more profound impact on mental health. Taken together, child maltreatment significantly contributes to increasing the risk of suicide (Duprey et al., 2023), and specifically, the various forms of traumatic experiences could differentially affect different suicidal behaviors.

Finally, the internal consistency of the overall scale was excellent; all the inter-item correlations (except item 10, which belongs to the validity subscale) had values >0.3. Four of the five subscales obtained alpha values >0.8. However, the physical neglect factor had an alpha below 0.7, reflecting moderate internal homogeneity as reported in previous studies (Gerdner and Allgulander, 2009; Kim et al., 2011; Hernandez et al., 2013; Grassi-Oliveira et al., 2014; Karos et al., 2014; Charak et al., 2017; He et al., 2019; Behn et al., 2020; Petrikova et al., 2021). The most robust internal consistency was found for the sexual abuse subscale, in accordance with the existing literature (Gerdner and Allgulander, 2009; Dovran et al., 2013; Hernandez et al., 2013; Charak et al., 2017; Sacchi et al., 2018; Kongerslev et al., 2019; Behn et al., 2020; Petrikova et al., 2021).

A primary strength of this study lies in its clinical significance and immediate practical relevance. Our research emphasizes how the CTQ-SF can play a vital role in clinical settings, aiding in the identification and understanding of childhood maltreatment among adolescents with suicide attempts. “Another strong aspect of this study is the representativeness of our sample, as the patients were recruited in seven different regions of Spain, comprising most of the national territory. Furthermore, the non-restrictive inclusion and exclusion criteria allowed the inclusion of patients with diverse clinical and demographic characteristics.” However, subsequent studies could be performed in different populations to further increase the validity and utility of the CTQ-SF. Some limitations should be taken into consideration. Firstly, the present study does not provide information about the convergent validity of the Spanish CTQ-SF. However, we did not find a suitable option available in Spanish to assess convergent validity. Second, child maltreatment explored through a self-report questionnaire was not contrasted with other objective evidence (such as pediatric or legal reports), leading to the possibility of participant memory and response bias. Third, a notable limitation of this study is the significant sex imbalance within the participant sample, with 87.0% of the adolescents being female. While this sex distribution may align with the specific context of the study population, the overrepresentation of females limits the extent to which the study’s results can be applied to a more sex-balanced population. Furthermore, although the sample size is representative, it is only fair for confirmatory factor analysis.

In conclusion, the Spanish version of the CTQ-SF is reliable and valid for measuring traumatic experiences in Spanish adolescents with at least one suicide attempt. As a self-reported instrument, it appears suitable for identifying childhood maltreatment in this population in routine clinical practice. Such identification can benefit patients and clinicians when making treatment plans, including assessment, treatment alternatives, and prognosis. Moreover, it is a feasible option as it does not require much time to administer, and the information obtained should be considered complementary to the clinician’s point of view.

## Data availability statement

The raw data supporting the conclusions of this article will be made available by the authors, without undue reservation.

## Ethics statement

The studies involving humans were approved by Clinical Research Ethics Committee at each of the participating sites [Hospital Clinic, IDIBAPS (PI19/000954), Hospital Universitario Parc Taulí (PI19/01484), Hospital Clínico San Carlos (PI19/01256), La Paz Institute for Health Research (PI19/00941), Hospital Universitario Central de Asturias (HUCA) (PI19/01027) (PI23/01277). Hospital Universitario Araba-Santiago (PI19/00569), and Hospital Universitario Virgen del Rocío (PI19/00685)]. It follows national and international guidelines: the latest version of the World Medical Association (2013), developed as ethical principles for medical research involving human subjects, and the International Council for Harmonization (ICH) Good Clinical Practice Guideline, GCP Directive 2005/28/EC. This study also complies with current Spanish legislation: Organic Law 3/2018 of December 5 on the Protection of Personal Data and Guarantee of Digital Rights and Regulation (EU) 2016/679 of the European Parliament and of the Council, version of April 27, 2016, known as the General Data Protection Regulation (GDPR). All participants and their parents or legal guardians gave their written informed consent before enrolment. The studies were conducted in accordance with the local legislation and institutional requirements. Written informed consent for participation in this study was provided by the participants’ legal guardians/next of kin.

## Author contributions

AG-F: Conceptualization, Methodology, Visualization, Writing – original draft, Writing – review & editing. CM-C: Formal analysis, Methodology, Writing – review & editing. AS-F-Q: Data curation, Formal analysis, Methodology, Software, Visualization, Writing – original draft, Writing – review & editing. TB-B: Supervision, Writing – review & editing. JA-J: Visualization, Writing – review & editing. WA-A: Visualization, Writing – review & editing. AC: Visualization, Writing – review & editing. MD-M: Visualization, Writing – review & editing. NG-T: Visualization, Writing – review & editing. SG: Visualization, Writing – review & editing. AG-P: Visualization, Writing – review & editing. IG: Visualization, Writing – review & editing. NI: Visualization, Writing – review & editing. KM: Visualization, Writing – review & editing. DP: Visualization, Writing – review & editing. IP-D: Visualization, Writing – review & editing. NR: Visualization, Writing – review & editing. MR-V: Visualization, Writing – review & editing. AT-L: Visualization, Writing – review & editing. IZ: Visualization, Writing – review & editing. VP: Visualization, Writing – review & editing. PS: Conceptualization, Supervision, Visualization, Writing – review & editing. MPG-P: Conceptualization, Formal analysis, Methodology, Validation, Visualization, Writing – review & editing.

## SURVIVE Group members

Íñigo Alberdi-Páramo, Margarita Alcami, Lorenzo Bracco, Maria Fe Bravo-Ortiz, Manuel Canal Rivero, Laura Comendador, Benedicto Crespo-Facorro, Cristina Diaz, Fernando Corbalán, Jennifer Fernández-Fernández, Eduardo Fernández-Jiménez, Veronica Fernandez-Rodrigues, Adriana Garcia-Ramos, Luis Jiménez-Treviño, Elvira Lara, Itziar Leal-Leturia, Maria Purificación Lopez-Peña, Lorea Mar-Barrutia, Julen Marin, Pablo Mola, Marta Navas, Luis Olivares, Angela Palao-Tarrero, Joaquín Punti, Pablo Reguera, Julia Rider, Carlamarina Rodríguez-Pereira, Maria Dolores Saiz Gónzalez, Yolanda Sanchez-Carro, Elisa Seijo-Zazo, Mireia Vázquez, Emma Vidal Bermejo, Eduard Vieta.
