# Supplementary material for: Validation of the Spanish Childhood Trauma Questionnaire-Short Form in adolescents with suicide attempts
Source: Front Psychol. 2024 Jul 9;15:1378486. doi: 10.3389/fpsyg.2024.1378486 (PMC11264239; doi:10.3389/fpsyg.2024.1378486)
Supplement: Supplementary file 1 [file Data_Sheet_1.pdf]

Supplementary table 1. Severity of Childhood Maltreatment.

	Frequency of childhood maltreatment severity by subtype			
	None n(%)	Low n(%)	Moderate n(%)	Extreme n(%)
Emotional Abuse	32 (15.4%)	36 (17.3%)	36 (17.3%)	104 (50.0%)
Physical Abuse	119 (57.2%)	30 (14.4%)	18 (8.7%)	41 (19.7%)
Sexual Abuse	112 (53.8%)	25 (12.0%)	25 (12.0%)	46 (22.1%)
Emotional Neglect	52 (25.0%)	70 (33.7%)	34 (16.3%)	52 (25.0%)
Physical Neglect	110 (52.9%)	38 (18.3%)	36 (17.3%)	24 (11.5%)

Note. CTQ-SF manual cut off scores for none, low, moderate, and extreme severity of childhood maltreatment: emotional abuse, ≤8, 9-12, 13-15, ≥16; physical abuse, ≤7, 8-9, 10-12, ≥13; sexual abuse, 5, 6-7, 8-12, ≥13; physical neglect: 5-7, 8-9, 10-12, ≥13; emotional neglect, 5-9, 10-14, 15-17, ≥18.

Supplementary Table 2. Component correlation matrix.

	Emotional Neglect	Sexual Abuse	Physical Abuse	Emotional Abuse	Physical Neglect
Emotional Neglect	1				
Sexual Abuse	0.346*	1			
Physical Abuse	0.462*	0.468*	1		
Emotional Abuse	0.679*	0.542*	0.732*	1	
Physical Neglect	0.857*	0.514*	0.666*	0.705*	1

* P(>|z|) = < .05

Supplementary Table 3. Descriptive analysis of the CTQ-SF items.

Items	Mean	SD	Median	Min	Max	Range	Skew	Kurtosis	SE
ITEM 1	1.380	0.825	1	1	5	4	2.540	6.591	0.057
ITEM 2R	2.337	1.297	2	1	5	4	0.617	-0.748	0.090
ITEM 3	3.043	1.405	3	1	5	4	-0.087	-1.261	0.097
ITEM 4	1.519	1.081	1	1	5	4	2.063	3.131	0.075
ITEM 5R	2.688	1.485	2	1	5	4	0.287	-1.357	0.103
ITEM 6	1.399	0.839	1	1	5	4	2.171	4.124	0.058
ITEM 7R	2.635	1.293	3	1	5	4	0.137	-1.201	0.090
ITEM 8	3.000	1.448	3	1	5	4	0.010	-1.357	0.100
ITEM 9	1.370	0.969	1	1	5	4	2.695	6.276	0.067
ITEM 10R	3.36	1.31	3	1	5	4	-0.14	-1.17	0.09
ITEM 11	2.067	1.402	1	1	5	4	1.000	-0.446	0.097
ITEM 12	1.798	1.329	1	1	5	4	1.441	0.627	0.092
ITEM 13R	2.423	1.309	2	1	5	4	0.476	-0.976	0.091
ITEM 14	2.788	1.439	3	1	5	4	0.158	-1.308	0.100
ITEM 15	1.962	1.303	1	1	5	4	1.113	-0.089	0.090
ITEM 16R	3.87	1.2	4	1	5	4	-0.83	-0.27	0.08
ITEM 17	1.466	1.125	1	1	5	4	2.386	4.309	0.078
ITEM 18	3.269	1.492	3	1	5	4	-0.299	-1.320	0.103
ITEM 19R	2.668	1.308	3	1	5	4	0.277	-1.088	0.091
ITEM 20	1.909	1.385	1	1	5	4	1.248	0.048	0.096
ITEM 21	1.486	1.133	1	1	5	4	2.264	3.743	0.079
ITEM 22R	3.56	1.31	4	1	5	4	-0.47	-0.9	0.09
ITEM 23	1.692	1.334	1	1	5	4	1.653	1.108	0.092
ITEM 24	1.865	1.458	1	1	5	4	1.330	0.106	0.101
ITEM 25	3.168	1.534	3	1	5	4	-0.202	-1.451	0.106
ITEM 26R	1.731	1.140	1	1	5	4	1.433	0.877	0.079
ITEM 27	1.798	1.407	1	1	5	4	1.447	0.447	0.098
IREM 28R	3.082	1.303	3	1	5	4	-0.164	-1.095	0.090

Note. SD: Standard Deviation; Min: Minimum; Max: Maximum; SE: Standard Error.

Supplementary Table 4. MD scale's frequency.

MD Score	Frequency	%
0	171	82.2
1	26	12.5
2	9	4.33
3	2	0.96

Note. MD: Minimization/Denial Scale.

Supplementary Table 5. MD scale t test for gender and age.

		Mean	t (df)	p
Gender	Male	0.33	0.93 (206)	0.184
	Female	0.23		
Age	≤15	0.22	-0.64 (206)	0.263
	>15	0.27		

Note. MD: Minimization/Denial Scale; df: degrees of freedom.

Supplementary Table 6. PA’s results.

Empiric eigenvalues	Simulated eigenvalues	95 percentile simulated eigenvalues
8.364	1.695	1.785
2.999	1.567	1.621
2.144	1.481	1.542
1.41	1.415	1.449
1.066	1.363	1.403

Note. PA: Parallel Analysis.

Supplementary Table 7. Fit indices for EFA CTQ-SF models.

Model	df	χ^2 (p-valor)	χ^2/df	RMSEA	SRMR	Δ X2	Δ df	p-Δ X2	TLI
6 factors	165	101.3446 (1)	0.61	0.054	0.028	-	-	-	0.928
5 factors	185	128.8844 (0.999)	0.7	0.055	0.032	27.5398	20	0.121	0.923
4 factors	206	193.4478 (0.725)	0.94	0.067	0.039	64.5634	21	< 0.01	0.888
3 factors	228	333.6566 (0)	1.46	0.078	0.052	140.2088	22	< 0.01	0.848

Note. EFA: Exploratory Factor Analysis; df: degrees of freedom; RMSEA: Root Mean Square Error of Approximation; SRMR: Standardised Root Mean Square Residual; Δ X2: increase of chi-square; Δ df: increase of degrees of freedom; p-Δ X2: increase of p value; TLI: Tucker-Lewis Index.

Supplementary Table 8. EFA’s Pattern Matrix.

Items	Sexual Abuse	Physical Abuse	Emotional Abuse	Emotional Neglect	Physical Neglect
ITEM 1	0.032	0.422	-0.167	0.012	0.4
ITEM 2R	0.075	-0.008	0.093	0.534	0.141
ITEM 3	-0.03	0.036	0.714	0.021	0.076
ITEM 4	0.195	0.243	-0.124	0.018	0.319
ITEM 5R	-0.007	0.05	0.025	0.719	-0.03
ITEM 6	0.162	0.281	-0.005	-0.072	0.283
ITEM 7R	0.103	0.068	0.045	0.649	0.217
ITEM 8	0.114	0.14	0.293	0.249	0.073
ITEM 9	0.021	0.66	0.077	-0.255	0.176
ITEM 11	0.018	0.863	0.105	0.094	-0.12
ITEM 12	0.041	0.649	-0.065	0.261	-0.055
ITEM 13R	0.045	-0.04	0.094	0.153	0.673
ITEM 14	-0.003	0.11	0.824	-0.029	-0.009
ITEM 15	0.06	0.575	0.216	0.089	-0.014
ITEM 17	0.017	0.564	0.12	-0.204	0.153
ITEM 18	0.117	-0.013	0.671	0.102	0.017
ITEM 19R	-0.01	-0.076	0.157	0.186	0.6
ITEM 20	0.841	-0.017	0.106	0.018	-0.05
ITEM 21	0.708	0.053	-0.016	0.065	-0.051
ITEM 23	0.904	-0.083	0.05	-0.028	-0.011
ITEM 24	0.834	0.046	-0.017	0.033	0.007
ITEM 25	0.285	0.083	0.4	0.04	0.074
ITEM 26R	0.084	0.267	-0.062	0.313	0.142
ITEM 27	0.91	0.03	-0.093	-0.041	0.06
ITEM 28R	-0.105	0	0.259	0.506	0.3

Note. EFA: Exploratory Factor Analysis

Supplementary Table 9. PCA's Pattern Matrix.

Items	Sexual Abuse	Physical Abuse	Emotional Abuse	Emotional Neglect	Physical Neglect
ITEM 1	0.029	0.418	-0.167	0.014	0.419
ITEM 2R	0.076	-0.008	0.09	0.546	0.123
ITEM 3	-0.03	0.043	0.708	0.026	0.069
ITEM 4	0.193	0.234	-0.115	0.024	0.319
ITEM 5R	-0.007	0.053	0.012	0.738	-0.054
ITEM 6	0.161	0.278	-0.003	-0.079	0.307
ITEM 7R	0.103	0.065	0.042	0.662	0.201
ITEM 8	0.113	0.139	0.294	0.246	0.071
ITEM 9	0.022	0.649	0.084	-0.244	0.176
ITEM 11	0.019	0.857	0.104	0.089	-0.112
ITEM 12	0.041	0.644	-0.064	0.256	-0.051
ITEM 13R	0.046	-0.052	0.112	0.183	0.64
ITEM 14	-0.003	0.121	0.81	-0.028	-0.008
ITEM 15	0.056	0.587	0.213	0.094	-0.023
ITEM 17	0.016	0.56	0.124	-0.196	0.153
ITEM 18	0.115	-0.012	0.683	0.096	0.009
ITEM 19R	-0.008	-0.086	0.173	0.211	0.568
ITEM 20	0.843	-0.014	0.105	0.017	-0.05
ITEM 21	0.715	0.056	-0.023	0.068	-0.052
ITEM 23	0.917	-0.088	0.05	-0.031	-0.01
ITEM 24	0.824	0.051	-0.018	0.035	0.009
ITEM 25	0.282	0.088	0.398	0.043	0.071
ITEM 26R	0.084	0.266	-0.066	0.32	0.14
ITEM 27	0.905	0.032	-0.092	-0.038	0.062
ITEM 28R	-0.106	-0.007	0.263	0.53	0.279

Note. PCA: Principal Component Analysis.

Supplementary Figure 1. Parallell Analysis and Scree Plot.

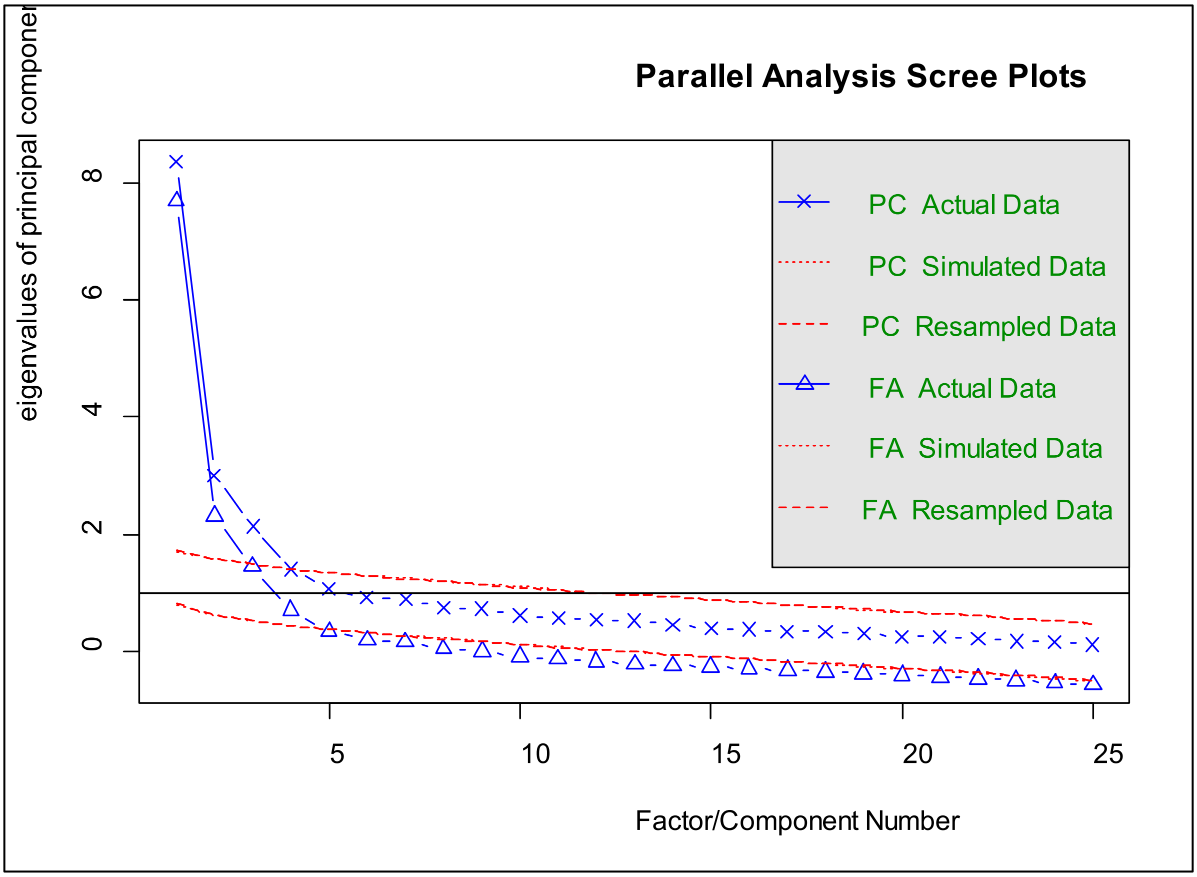

Note. PC: Principal Component; FA: Factor Analysis
